# 3β,11α-Dihy­droxy-12-ursen-3-yl palmitate

**DOI:** 10.1107/S1600536811047106

**Published:** 2011-11-19

**Authors:** Yong-Liang Yuan, Su-Ping Bai, Hui-Juan Liang, Dan-Dan Ye

**Affiliations:** a School of Pharmacy, Xinxiang Medical University, Xinxiang, Henan 453000, People’s Republic of China

## Abstract

In the title compound, C_46_H_80_O_3_, a natural ursane-type triperpenoid, four of the five six-membered rings adopt chair conformations; the fifth, which has a C=C double bond, adopts an approximate half-boat conformation. In the crystal, mol­ecules are linked by O—H⋯O hydrogen bonds, forming chains along [010].

## Related literature

For standard bond lengths, see: Allen *et al.* (1987 ▶). For spectroscopic properties of the title compound, see: Kakuda *et al.* (2003 ▶).
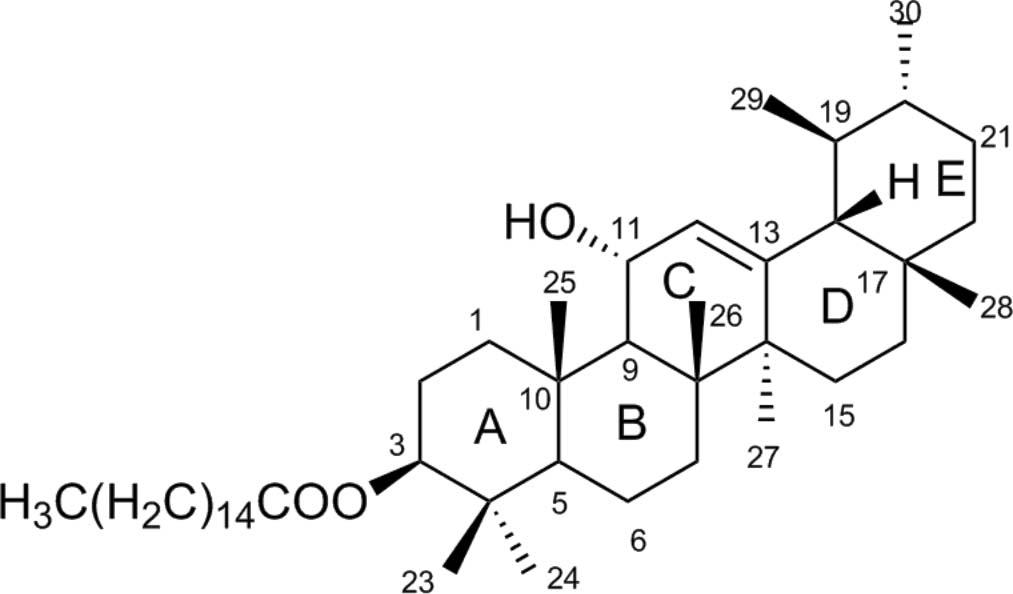


## Experimental

### 

#### Crystal data

C_46_H_80_O_3_
*M* *_r_* = 681.10
Monoclinic, 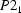

*a* = 11.389 (2) Å
*b* = 15.714 (3) Å
*c* = 11.766 (2) Å
β = 98.925 (3)°
*V* = 2080.3 (7) Å^3^
*Z* = 2
Mo *K*α radiation
μ = 0.07 mm^−1^
*T* = 93 K
0.60 × 0.50 × 0.30 mm

#### Data collection

Rigaku AFC10/Saturn724+ diffractometer
16995 measured reflections
4832 independent reflections
4397 reflections with *I* > 2σ(*I*)
*R*_int_ = 0.032

#### Refinement

*R*[*F* ^2^ > 2σ(*F* ^2^)] = 0.043
*wR*(*F* ^2^) = 0.098
*S* = 1.00
4832 reflections
455 parameters
1 restraint
H atoms treated by a mixture of independent and constrained refinement
Δρ_max_ = 0.22 e Å^−3^
Δρ_min_ = −0.15 e Å^−3^

### 

Data collection: *CrystalClear* (Rigaku, 2008 ▶); cell refinement: *CrystalClear*; data reduction: *CrystalClear*; program(s) used to solve structure: *SHELXS97* (Sheldrick, 2008 ▶); program(s) used to refine structure: *SHELXL97* (Sheldrick, 2008 ▶); molecular graphics: *SHELXTL* (Sheldrick, 2008 ▶) and *PLATON* (Spek, 2009 ▶); software used to prepare material for publication: *SHELXTL*.

## Supplementary Material

Crystal structure: contains datablock(s) global, I. DOI: 10.1107/S1600536811047106/bx2380sup1.cif

Structure factors: contains datablock(s) I. DOI: 10.1107/S1600536811047106/bx2380Isup2.hkl

Additional supplementary materials:  crystallographic information; 3D view; checkCIF report

## Figures and Tables

**Table 1:** Hydrogen-bond geometry (Å, °)

*D*—H⋯*A*	*D*—H	H⋯*A*	*D*⋯*A*	*D*—H⋯*A*
O1—H1*O*⋯O3^i^	0.79 (4)	2.13 (4)	2.886 (3)	161 (3)

Symmetry code: (i) 
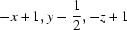
.

**Fig. 1.**
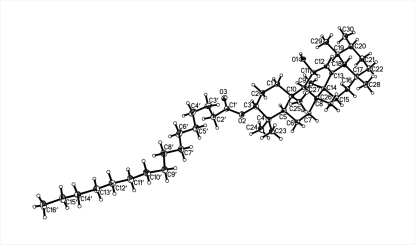
A view of the molecular structure of compound (I). Displacement ellipsoids are drawn at the 50% probability level.

**Fig. 2.**
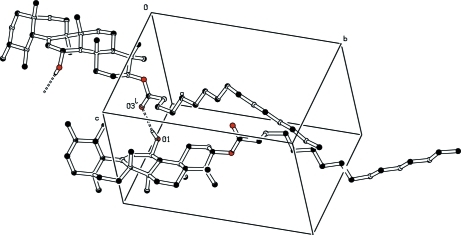
The crystal packing of (I), viewed along the a axis, showing the O—H···O hydrogen bonds as dashed lines.Symmetry code: (i) -x+1, y-1/2, -z+1.

## supplementary crystallographic information

### Comment

The title compound, (I), is a naturally occurring ursene-type triterpene isolated from the medicinal plant Saussurea nivea Turcz. This plant has been used as antibacterial, inflammation-diminishing drugs and febrifuge. The structure of compound (I) has been reported previously based on spectroscopic methods (Kakuda, *et al.*, 2003). In order to further confirm the structure and conformation of (I), a crystal structure analysis, reported here, was undertaken.

The X-ray crystallographic analysis of (I) confirms the previously proposed molecular structure of (I) as an ursane-type triterpene. Fig. 1 shows its conformation: the palmitoyl group and the hydroxyl group is connected to C3 and C11 in β and α-orientation, respectively; while the Me-20 and Me-30 adopted β and α-orientation at C19 and C20, respectively; and the double bond is located at C12 and C13.The molecule contains five six-membered rings. The A/B and B/C ring junctions show *trans* fusion and the geometry of the rings is *cis* at the D/E ring junction. The bond lengths and angles of (I) have normal values (Allen *et al.*, 1987), with the following average values (Å): C*sp*^3^—C*sp*^3^ = 1.533 (3), C*sp*^3^—C*sp*^2^ = 1.522 (3), C*sp*^2^—C*sp*^2^ = 1.333 (3), C═O = 1.211 (3), C*sp*^2^—O = 1.330(3, C*sp*^3^—O = 1.452 (3)). Rings A, B and E have slightly flattened chair conformations, with average torsion angles of 53.6 (3), 53.4 (3) and 54.2 °, respectively. Chair conformations of ring D are twisted, with torsion angle range from 34.1 to 63.0 °. Ring C adopts an approximate half-boat conformation. The long carbon chain connected to C3, which takes anti-conformations in C4'-C9' and C10'-C16' with average dihedral angles of 176.9 and 177.0 °, has two turns on C3'-C4' and C9'-C10' in *gauche*-conformations with torsion angles of 73.9 and 69.4°, respectively. The crystal packing is stabilized by intermolecular O—H···O hydrogen bonds involving the carbonyl and hydroxyl groups (Table1 and Fig.2). The hydrogen bonds link the molecules into chains along the b axis.

### Experimental

The dried and crushed leaves of Saussurea nivea Turcz (10 kg, collected from Tongbai Mountain, Henan Province, China) were extracted three times with Me_2_CO at room temperature for seven days. The extract was filtered and the solvent was removed under reduced pressure. The residue was partitioned between water and ethylacetate. After removing the solvent, the ethyl acetate residue was separated by repeated silica gel (200–300 mesh) column chromatography and recrystallization in CHCl_3_/Me_2_CO (30:1) to afford 20 mg of compound (I) (Optical rotation: [α]_D_^25^ +31.1 ° (c 0.27,CHCl_3_). Crystals suitable for x-ray experiment were obtained by slow evaporation of a solution of the compound (I) in Me_2_CO at room temperature.

### Refinement

All H atoms were included in calculated position and refined as riding atoms,with C—H = 0.95Å (CH_3_), 0.93 and 0.97 Å(CH_2_), 0.98 Å(CH), and with *U*_iso_(H) =1.2*U*_eq_(C). In the absence of significant anomalous scattering effects,Friedel pairs were merged. The choice of enantiomer was based on comparison of the optical rotation with that of related compounds with known stereochemistry.

### Crystal data

**Table:**

C_46_H_80_O_3_	*F*(000) = 760
*M**_r_* = 681.10	*D*_x_ = 1.087 Mg m^−^^3^
Monoclinic, *P*2_1_	Mo *K*α radiation, λ = 0.71073 Å
*a* = 11.389 (2) Å	Cell parameters from 7345 reflections
*b* = 15.714 (3) Å	θ = 3.0–27.5°
*c* = 11.766 (2) Å	µ = 0.07 mm^−^^1^
β = 98.925 (3)°	*T* = 93 K
*V* = 2080.3 (7) Å^3^	Block, colourless
*Z* = 2	0.60 × 0.50 × 0.30 mm

### Data collection

**Table:**

Rigaku AFC10/Saturn724+ diffractometer	4397 reflections with *I* > 2σ(*I*)
Radiation source: Rotating Anode	*R*_int_ = 0.032
graphite	θ_max_ = 27.5°, θ_min_ = 3.1°
Detector resolution: 28.5714 pixels mm^-1^	*h* = −14→14
phi and ω scans	*k* = −20→20
16995 measured reflections	*l* = −14→14
4832 independent reflections	

### Refinement

**Table:**

Refinement on *F*^2^	Primary atom site location: structure-invariant direct methods
Least-squares matrix: full	Secondary atom site location: difference Fourier map
*R*[*F*^2^ > 2σ(*F*^2^)] = 0.043	Hydrogen site location: inferred from neighbouring sites
*wR*(*F*^2^) = 0.098	H atoms treated by a mixture of independent and constrained refinement
*S* = 1.00	*w* = 1/[σ^2^(*F*_o_^2^) + (0.0286*P*)^2^ + 0.860*P*] where *P* = (*F*_o_^2^ + 2*F*_c_^2^)/3
4832 reflections	(Δ/σ)_max_ < 0.001
455 parameters	Δρ_max_ = 0.22 e Å^−^^3^
1 restraint	Δρ_min_ = −0.15 e Å^−^^3^

### Special details

**Table:**

Geometry. All e.s.d.'s (except the e.s.d. in the dihedral angle between two l.s. planes) are estimated using the full covariance matrix. The cell e.s.d.'s are taken into account individually in the estimation of e.s.d.'s in distances, angles and torsion angles; correlations between e.s.d.'s in cell parameters are only used when they are defined by crystal symmetry. An approximate (isotropic) treatment of cell e.s.d.'s is used for estimating e.s.d.'s involving l.s. planes.
Refinement. Refinement of *F*^2^ against ALL reflections. The weighted *R*-factor *wR* and goodness of fit *S* are based on *F*^2^, conventional *R*-factors *R* are based on *F*, with *F* set to zero for negative *F*^2^. The threshold expression of *F*^2^ > σ(*F*^2^) is used only for calculating *R*-factors(gt) *etc*. and is not relevant to the choice of reflections for refinement. *R*-factors based on *F*^2^ are statistically about twice as large as those based on *F*, and *R*- factors based on ALL data will be even larger.

### Fractional atomic coordinates and isotropic or equivalent isotropic displacement parameters (Å^2^)

**Table:**

	*x*	*y*	*z*	*U*_iso_*/*U*_eq_	
O1	0.7358 (2)	0.09757 (13)	0.56526 (16)	0.0423 (5)	
O2	0.66023 (16)	0.51467 (12)	0.64282 (16)	0.0373 (4)	
O3	0.48079 (18)	0.51257 (14)	0.53336 (17)	0.0469 (5)	
C1	0.7086 (2)	0.27759 (15)	0.6092 (2)	0.0282 (5)	
H1A	0.7494	0.2468	0.5531	0.034*	
H1B	0.6248	0.2584	0.5980	0.034*	
C2	0.7121 (2)	0.37373 (16)	0.5849 (2)	0.0321 (6)	
H2A	0.7958	0.3925	0.5903	0.039*	
H2B	0.6716	0.3853	0.5057	0.039*	
C3	0.6522 (2)	0.42345 (17)	0.6692 (2)	0.0328 (6)	
H3	0.5664	0.4069	0.6590	0.039*	
C4	0.7061 (2)	0.41063 (16)	0.7958 (2)	0.0302 (5)	
C5	0.7115 (2)	0.31251 (16)	0.8183 (2)	0.0263 (5)	
H5	0.6264	0.2944	0.8085	0.032*	
C6	0.7608 (2)	0.28945 (16)	0.9424 (2)	0.0298 (5)	
H6A	0.7300	0.3299	0.9952	0.036*	
H6B	0.8485	0.2940	0.9545	0.036*	
C7	0.7248 (2)	0.19890 (16)	0.9696 (2)	0.0286 (5)	
H7A	0.6374	0.1967	0.9651	0.034*	
H7B	0.7605	0.1850	1.0496	0.034*	
C8	0.76315 (19)	0.13074 (16)	0.8885 (2)	0.0245 (5)	
C9	0.73602 (19)	0.15932 (16)	0.76015 (19)	0.0238 (5)	
H9	0.6476	0.1558	0.7394	0.029*	
C10	0.7685 (2)	0.25440 (16)	0.7329 (2)	0.0253 (5)	
C11	0.7853 (2)	0.09126 (17)	0.6844 (2)	0.0288 (5)	
H11	0.8727	0.1016	0.6903	0.035*	
C12	0.7700 (2)	0.00159 (16)	0.7251 (2)	0.0274 (5)	
H12	0.7920	−0.0427	0.6775	0.033*	
C13	0.72899 (19)	−0.02268 (15)	0.8202 (2)	0.0241 (5)	
C14	0.6930 (2)	0.04465 (16)	0.9029 (2)	0.0251 (5)	
C15	0.7157 (2)	0.01434 (17)	1.0297 (2)	0.0307 (5)	
H15A	0.7993	0.0270	1.0626	0.037*	
H15B	0.6641	0.0473	1.0740	0.037*	
C16	0.6926 (2)	−0.08099 (17)	1.0454 (2)	0.0318 (5)	
H16A	0.6064	−0.0922	1.0249	0.038*	
H16B	0.7164	−0.0962	1.1273	0.038*	
C17	0.7605 (2)	−0.13792 (17)	0.9712 (2)	0.0293 (5)	
C18	0.7145 (2)	−0.11755 (16)	0.8435 (2)	0.0268 (5)	
H18	0.7681	−0.1483	0.7976	0.032*	
C19	0.5867 (2)	−0.15270 (16)	0.8029 (2)	0.0288 (5)	
H19	0.5315	−0.1230	0.8482	0.035*	
C20	0.5810 (2)	−0.24905 (17)	0.8281 (2)	0.0320 (5)	
H20	0.6385	−0.2786	0.7854	0.038*	
C21	0.6193 (2)	−0.26572 (17)	0.9567 (2)	0.0350 (6)	
H21A	0.5630	−0.2375	1.0008	0.042*	
H21B	0.6170	−0.3276	0.9717	0.042*	
C22	0.7438 (2)	−0.23238 (18)	0.9970 (2)	0.0351 (6)	
H22A	0.8006	−0.2661	0.9596	0.042*	
H22B	0.7638	−0.2414	1.0810	0.042*	
C23	0.8264 (2)	0.45627 (17)	0.8259 (2)	0.0326 (6)	
H23A	0.8791	0.4389	0.7716	0.039*	
H23B	0.8628	0.4410	0.9042	0.039*	
H23C	0.8142	0.5180	0.8209	0.039*	
C24	0.6193 (2)	0.45184 (18)	0.8686 (3)	0.0413 (7)	
H24A	0.6090	0.5122	0.8485	0.050*	
H24B	0.6516	0.4465	0.9505	0.050*	
H24C	0.5422	0.4229	0.8531	0.050*	
C25	0.9039 (2)	0.26877 (17)	0.7378 (2)	0.0289 (5)	
H25A	0.9380	0.2211	0.7003	0.035*	
H25B	0.9420	0.2725	0.8182	0.035*	
H25C	0.9172	0.3218	0.6979	0.035*	
C26	0.8995 (2)	0.11656 (17)	0.9255 (2)	0.0294 (5)	
H26A	0.9419	0.1698	0.9165	0.035*	
H26B	0.9271	0.0725	0.8770	0.035*	
H26C	0.9150	0.0985	1.0062	0.035*	
C27	0.5556 (2)	0.05609 (17)	0.8704 (2)	0.0296 (5)	
H27A	0.5349	0.0629	0.7869	0.036*	
H27B	0.5304	0.1067	0.9090	0.036*	
H27C	0.5152	0.0058	0.8949	0.036*	
C28	0.8948 (2)	−0.11911 (19)	0.9984 (2)	0.0394 (6)	
H28A	0.9383	−0.1596	0.9570	0.047*	
H28B	0.9215	−0.1248	1.0813	0.047*	
H28C	0.9101	−0.0610	0.9741	0.047*	
C29	0.5451 (3)	−0.13413 (19)	0.6761 (2)	0.0426 (7)	
H29A	0.5984	−0.1620	0.6298	0.051*	
H29B	0.5458	−0.0725	0.6631	0.051*	
H29C	0.4642	−0.1559	0.6537	0.051*	
C30	0.4576 (2)	−0.28749 (19)	0.7892 (2)	0.0398 (6)	
H30A	0.4577	−0.3473	0.8127	0.048*	
H30B	0.4379	−0.2836	0.7053	0.048*	
H30C	0.3985	−0.2561	0.8248	0.048*	
C1'	0.5716 (2)	0.54980 (18)	0.5713 (2)	0.0350 (6)	
C2'	0.5993 (3)	0.64035 (18)	0.5409 (2)	0.0390 (6)	
H2'1	0.5272	0.6759	0.5379	0.047*	
H2'2	0.6615	0.6639	0.6007	0.047*	
C3'	0.6418 (3)	0.6424 (2)	0.4257 (3)	0.0492 (8)	
H3'1	0.5834	0.6122	0.3687	0.059*	
H3'2	0.7181	0.6113	0.4320	0.059*	
C4'	0.6596 (3)	0.7331 (2)	0.3812 (3)	0.0549 (9)	
H4'1	0.6651	0.7296	0.2982	0.066*	
H4'2	0.5883	0.7672	0.3891	0.066*	
C5'	0.7677 (3)	0.77948 (19)	0.4411 (2)	0.0403 (6)	
H5'1	0.8392	0.7444	0.4376	0.048*	
H5'2	0.7601	0.7874	0.5231	0.048*	
C6'	0.7836 (3)	0.8663 (2)	0.3869 (3)	0.0511 (8)	
H6'1	0.7864	0.8577	0.3040	0.061*	
H6'2	0.7125	0.9012	0.3930	0.061*	
C7'	0.8925 (2)	0.91628 (18)	0.4381 (2)	0.0369 (6)	
H7'1	0.9642	0.8808	0.4367	0.044*	
H7'2	0.8875	0.9296	0.5195	0.044*	
C8'	0.9052 (3)	0.99922 (19)	0.3730 (3)	0.0407 (6)	
H8'1	0.9087	0.9854	0.2915	0.049*	
H8'2	0.8333	1.0342	0.3750	0.049*	
C9'	1.0140 (3)	1.05190 (18)	0.4201 (3)	0.0413 (7)	
H9'1	1.0856	1.0156	0.4244	0.050*	
H9'2	1.0071	1.0704	0.4992	0.050*	
C10'	1.0300 (2)	1.13012 (18)	0.3475 (3)	0.0414 (7)	
H10A	1.0274	1.1123	0.2665	0.050*	
H10B	1.1096	1.1547	0.3740	0.050*	
C11'	0.9365 (3)	1.19878 (19)	0.3527 (3)	0.0424 (7)	
H11A	0.9364	1.2146	0.4341	0.051*	
H11B	0.8573	1.1750	0.3225	0.051*	
C12'	0.9563 (2)	1.27902 (18)	0.2846 (2)	0.0382 (6)	
H12A	1.0346	1.3038	0.3156	0.046*	
H12B	0.9574	1.2635	0.2032	0.046*	
C13'	0.8604 (2)	1.34523 (19)	0.2901 (3)	0.0396 (6)	
H13A	0.8564	1.3576	0.3719	0.048*	
H13B	0.7829	1.3208	0.2556	0.048*	
C14'	0.8783 (2)	1.42849 (18)	0.2295 (2)	0.0357 (6)	
H14A	0.9517	1.4562	0.2686	0.043*	
H14B	0.8894	1.4162	0.1493	0.043*	
C15'	0.7755 (3)	1.4889 (2)	0.2281 (3)	0.0464 (7)	
H15C	0.7619	1.4983	0.3082	0.056*	
H15D	0.7032	1.4618	0.1857	0.056*	
C16'	0.7929 (3)	1.5745 (2)	0.1735 (3)	0.0502 (8)	
H16'A	0.8613	1.6035	0.2178	0.060*	
H16'B	0.7215	1.6093	0.1733	0.060*	
H16'C	0.8072	1.5660	0.0943	0.060*	
H1O	0.674 (3)	0.074 (3)	0.554 (3)	0.057 (11)*	

### Atomic displacement parameters (Å^2^)

**Table:**

	*U*^11^	*U*^22^	*U*^33^	*U*^12^	*U*^13^	*U*^23^
O1	0.0667 (14)	0.0380 (11)	0.0214 (10)	−0.0177 (11)	0.0045 (9)	−0.0005 (8)
O2	0.0324 (9)	0.0323 (10)	0.0443 (11)	−0.0016 (8)	−0.0027 (8)	0.0066 (9)
O3	0.0447 (11)	0.0427 (11)	0.0469 (12)	0.0035 (10)	−0.0134 (9)	−0.0033 (10)
C1	0.0303 (12)	0.0304 (13)	0.0224 (12)	−0.0072 (10)	−0.0010 (9)	0.0018 (10)
C2	0.0325 (12)	0.0337 (14)	0.0284 (13)	−0.0080 (11)	−0.0006 (10)	0.0063 (11)
C3	0.0245 (12)	0.0316 (13)	0.0411 (15)	−0.0042 (10)	0.0009 (10)	0.0036 (12)
C4	0.0253 (12)	0.0293 (13)	0.0364 (14)	−0.0021 (10)	0.0057 (10)	−0.0010 (11)
C5	0.0221 (11)	0.0303 (12)	0.0267 (13)	−0.0026 (9)	0.0039 (9)	0.0001 (10)
C6	0.0312 (12)	0.0323 (13)	0.0261 (13)	−0.0021 (10)	0.0054 (10)	−0.0045 (10)
C7	0.0304 (12)	0.0346 (14)	0.0208 (12)	−0.0002 (10)	0.0044 (9)	0.0002 (10)
C8	0.0205 (10)	0.0308 (12)	0.0219 (12)	−0.0010 (9)	0.0026 (8)	0.0005 (10)
C9	0.0204 (10)	0.0305 (12)	0.0196 (11)	−0.0041 (9)	0.0005 (8)	0.0008 (9)
C10	0.0224 (11)	0.0291 (12)	0.0242 (12)	−0.0053 (9)	0.0031 (9)	0.0001 (10)
C11	0.0304 (12)	0.0333 (13)	0.0231 (12)	−0.0075 (10)	0.0057 (10)	−0.0007 (10)
C12	0.0264 (11)	0.0304 (13)	0.0262 (12)	−0.0026 (10)	0.0067 (9)	−0.0030 (10)
C13	0.0199 (10)	0.0300 (12)	0.0214 (12)	0.0007 (9)	0.0003 (9)	0.0020 (9)
C14	0.0224 (11)	0.0310 (12)	0.0223 (12)	0.0010 (9)	0.0047 (9)	0.0019 (9)
C15	0.0321 (12)	0.0385 (14)	0.0218 (12)	0.0015 (11)	0.0053 (10)	0.0030 (11)
C16	0.0321 (12)	0.0404 (14)	0.0224 (12)	0.0015 (11)	0.0027 (10)	0.0089 (11)
C17	0.0235 (11)	0.0346 (13)	0.0285 (13)	0.0035 (10)	0.0000 (9)	0.0103 (11)
C18	0.0240 (11)	0.0299 (13)	0.0271 (13)	0.0036 (9)	0.0059 (9)	0.0046 (10)
C19	0.0283 (12)	0.0307 (13)	0.0271 (13)	0.0011 (10)	0.0031 (9)	0.0049 (10)
C20	0.0317 (13)	0.0320 (13)	0.0329 (14)	−0.0004 (10)	0.0071 (10)	0.0066 (11)
C21	0.0369 (14)	0.0334 (14)	0.0355 (15)	0.0008 (11)	0.0081 (11)	0.0118 (11)
C22	0.0320 (13)	0.0382 (15)	0.0341 (14)	0.0056 (11)	0.0024 (11)	0.0119 (12)
C23	0.0313 (13)	0.0309 (13)	0.0349 (14)	−0.0039 (10)	0.0028 (10)	−0.0002 (11)
C24	0.0387 (15)	0.0358 (15)	0.0519 (18)	0.0037 (12)	0.0152 (13)	0.0013 (13)
C25	0.0236 (11)	0.0329 (13)	0.0306 (13)	−0.0055 (10)	0.0052 (9)	0.0002 (10)
C26	0.0235 (11)	0.0376 (14)	0.0256 (12)	−0.0009 (10)	−0.0006 (9)	0.0008 (10)
C27	0.0217 (11)	0.0323 (13)	0.0357 (14)	0.0027 (10)	0.0072 (10)	0.0030 (11)
C28	0.0277 (13)	0.0424 (16)	0.0453 (16)	0.0058 (12)	−0.0034 (11)	0.0110 (13)
C29	0.0504 (16)	0.0360 (15)	0.0366 (15)	−0.0131 (13)	−0.0082 (12)	0.0079 (12)
C30	0.0389 (15)	0.0412 (16)	0.0398 (16)	−0.0052 (12)	0.0074 (12)	0.0088 (13)
C1'	0.0362 (13)	0.0364 (14)	0.0313 (14)	0.0044 (11)	0.0018 (11)	−0.0012 (11)
C2'	0.0428 (15)	0.0351 (15)	0.0373 (15)	0.0054 (12)	0.0005 (12)	0.0027 (12)
C3'	0.0579 (19)	0.0502 (19)	0.0373 (17)	−0.0116 (15)	0.0007 (13)	−0.0043 (14)
C4'	0.057 (2)	0.066 (2)	0.0379 (17)	−0.0130 (17)	−0.0059 (14)	0.0150 (16)
C5'	0.0404 (15)	0.0463 (17)	0.0338 (15)	0.0011 (12)	0.0046 (11)	0.0078 (13)
C6'	0.0489 (17)	0.054 (2)	0.0472 (18)	−0.0057 (15)	−0.0046 (14)	0.0187 (15)
C7'	0.0400 (14)	0.0384 (15)	0.0336 (14)	0.0072 (12)	0.0104 (11)	0.0007 (12)
C8'	0.0456 (15)	0.0388 (15)	0.0388 (15)	0.0046 (13)	0.0101 (12)	0.0040 (12)
C9'	0.0404 (15)	0.0389 (15)	0.0439 (17)	0.0098 (12)	0.0046 (12)	−0.0022 (13)
C10'	0.0367 (14)	0.0356 (15)	0.0534 (18)	0.0005 (12)	0.0115 (13)	−0.0058 (14)
C11'	0.0398 (15)	0.0373 (15)	0.0516 (18)	0.0040 (12)	0.0114 (13)	0.0041 (13)
C12'	0.0379 (14)	0.0349 (15)	0.0420 (16)	−0.0005 (11)	0.0075 (12)	−0.0010 (12)
C13'	0.0332 (14)	0.0396 (15)	0.0454 (16)	−0.0024 (12)	0.0042 (12)	0.0044 (13)
C14'	0.0322 (13)	0.0344 (14)	0.0399 (15)	−0.0026 (11)	0.0036 (11)	0.0013 (12)
C15'	0.0385 (15)	0.0436 (17)	0.0577 (19)	0.0051 (13)	0.0095 (14)	0.0114 (14)
C16'	0.0411 (16)	0.0417 (17)	0.068 (2)	0.0077 (13)	0.0100 (15)	0.0155 (16)

### Geometric parameters (Å, °)

**Table:**

O1—C11	1.431 (3)	C24—H24B	0.9800
O1—H1O	0.79 (4)	C24—H24C	0.9800
O2—C1'	1.330 (3)	C25—H25A	0.9800
O2—C3	1.473 (3)	C25—H25B	0.9800
O3—C1'	1.211 (3)	C25—H25C	0.9800
C1—C2	1.539 (3)	C26—H26A	0.9800
C1—C10	1.552 (3)	C26—H26B	0.9800
C1—H1A	0.9900	C26—H26C	0.9800
C1—H1B	0.9900	C27—H27A	0.9800
C2—C3	1.507 (4)	C27—H27B	0.9800
C2—H2A	0.9900	C27—H27C	0.9800
C2—H2B	0.9900	C28—H28A	0.9800
C3—C4	1.533 (4)	C28—H28B	0.9800
C3—H3	1.0000	C28—H28C	0.9800
C4—C23	1.539 (3)	C29—H29A	0.9800
C4—C24	1.547 (4)	C29—H29B	0.9800
C4—C5	1.564 (3)	C29—H29C	0.9800
C5—C6	1.524 (3)	C30—H30A	0.9800
C5—C10	1.571 (3)	C30—H30B	0.9800
C5—H5	1.0000	C30—H30C	0.9800
C6—C7	1.528 (3)	C1'—C2'	1.513 (4)
C6—H6A	0.9900	C2'—C3'	1.508 (4)
C6—H6B	0.9900	C2'—H2'1	0.9900
C7—C8	1.542 (3)	C2'—H2'2	0.9900
C7—H7A	0.9900	C3'—C4'	1.542 (5)
C7—H7B	0.9900	C3'—H3'1	0.9900
C8—C9	1.559 (3)	C3'—H3'2	0.9900
C8—C26	1.562 (3)	C4'—C5'	1.508 (4)
C8—C14	1.594 (3)	C4'—H4'1	0.9900
C9—C11	1.552 (3)	C4'—H4'2	0.9900
C9—C10	1.584 (3)	C5'—C6'	1.528 (4)
C9—H9	1.0000	C5'—H5'1	0.9900
C10—C25	1.550 (3)	C5'—H5'2	0.9900
C11—C12	1.507 (4)	C6'—C7'	1.512 (4)
C11—H11	1.0000	C6'—H6'1	0.9900
C12—C13	1.333 (3)	C6'—H6'2	0.9900
C12—H12	0.9500	C7'—C8'	1.530 (4)
C13—C18	1.530 (3)	C7'—H7'1	0.9900
C13—C14	1.536 (3)	C7'—H7'2	0.9900
C14—C15	1.549 (3)	C8'—C9'	1.521 (4)
C14—C27	1.563 (3)	C8'—H8'1	0.9900
C15—C16	1.537 (4)	C8'—H8'2	0.9900
C15—H15A	0.9900	C9'—C10'	1.524 (4)
C15—H15B	0.9900	C9'—H9'1	0.9900
C16—C17	1.539 (4)	C9'—H9'2	0.9900
C16—H16A	0.9900	C10'—C11'	1.524 (4)
C16—H16B	0.9900	C10'—H10A	0.9900
C17—C22	1.533 (4)	C10'—H10B	0.9900
C17—C28	1.542 (3)	C11'—C12'	1.529 (4)
C17—C18	1.546 (3)	C11'—H11A	0.9900
C18—C19	1.561 (3)	C11'—H11B	0.9900
C18—H18	1.0000	C12'—C13'	1.517 (4)
C19—C29	1.522 (3)	C12'—H12A	0.9900
C19—C20	1.546 (4)	C12'—H12B	0.9900
C19—H19	1.0000	C13'—C14'	1.519 (4)
C20—C21	1.531 (4)	C13'—H13A	0.9900
C20—C30	1.533 (4)	C13'—H13B	0.9900
C20—H20	1.0000	C14'—C15'	1.505 (4)
C21—C22	1.517 (4)	C14'—H14A	0.9900
C21—H21A	0.9900	C14'—H14B	0.9900
C21—H21B	0.9900	C15'—C16'	1.517 (4)
C22—H22A	0.9900	C15'—H15C	0.9900
C22—H22B	0.9900	C15'—H15D	0.9900
C23—H23A	0.9800	C16'—H16'A	0.9800
C23—H23B	0.9800	C16'—H16'B	0.9800
C23—H23C	0.9800	C16'—H16'C	0.9800
C24—H24A	0.9800		
			
C11—O1—H1O	110 (3)	C4—C24—H24B	109.5
C1'—O2—C3	118.1 (2)	H24A—C24—H24B	109.5
C2—C1—C10	112.6 (2)	C4—C24—H24C	109.5
C2—C1—H1A	109.1	H24A—C24—H24C	109.5
C10—C1—H1A	109.1	H24B—C24—H24C	109.5
C2—C1—H1B	109.1	C10—C25—H25A	109.5
C10—C1—H1B	109.1	C10—C25—H25B	109.5
H1A—C1—H1B	107.8	H25A—C25—H25B	109.5
C3—C2—C1	111.0 (2)	C10—C25—H25C	109.5
C3—C2—H2A	109.4	H25A—C25—H25C	109.5
C1—C2—H2A	109.4	H25B—C25—H25C	109.5
C3—C2—H2B	109.4	C8—C26—H26A	109.5
C1—C2—H2B	109.4	C8—C26—H26B	109.5
H2A—C2—H2B	108.0	H26A—C26—H26B	109.5
O2—C3—C2	108.4 (2)	C8—C26—H26C	109.5
O2—C3—C4	107.7 (2)	H26A—C26—H26C	109.5
C2—C3—C4	114.7 (2)	H26B—C26—H26C	109.5
O2—C3—H3	108.6	C14—C27—H27A	109.5
C2—C3—H3	108.6	C14—C27—H27B	109.5
C4—C3—H3	108.6	H27A—C27—H27B	109.5
C3—C4—C23	111.7 (2)	C14—C27—H27C	109.5
C3—C4—C24	106.8 (2)	H27A—C27—H27C	109.5
C23—C4—C24	107.5 (2)	H27B—C27—H27C	109.5
C3—C4—C5	107.1 (2)	C17—C28—H28A	109.5
C23—C4—C5	114.2 (2)	C17—C28—H28B	109.5
C24—C4—C5	109.3 (2)	H28A—C28—H28B	109.5
C6—C5—C4	113.3 (2)	C17—C28—H28C	109.5
C6—C5—C10	110.3 (2)	H28A—C28—H28C	109.5
C4—C5—C10	118.3 (2)	H28B—C28—H28C	109.5
C6—C5—H5	104.4	C19—C29—H29A	109.5
C4—C5—H5	104.4	C19—C29—H29B	109.5
C10—C5—H5	104.4	H29A—C29—H29B	109.5
C5—C6—C7	110.6 (2)	C19—C29—H29C	109.5
C5—C6—H6A	109.5	H29A—C29—H29C	109.5
C7—C6—H6A	109.5	H29B—C29—H29C	109.5
C5—C6—H6B	109.5	C20—C30—H30A	109.5
C7—C6—H6B	109.5	C20—C30—H30B	109.5
H6A—C6—H6B	108.1	H30A—C30—H30B	109.5
C6—C7—C8	113.97 (19)	C20—C30—H30C	109.5
C6—C7—H7A	108.8	H30A—C30—H30C	109.5
C8—C7—H7A	108.8	H30B—C30—H30C	109.5
C6—C7—H7B	108.8	O3—C1'—O2	123.6 (3)
C8—C7—H7B	108.8	O3—C1'—C2'	124.4 (3)
H7A—C7—H7B	107.7	O2—C1'—C2'	112.0 (2)
C7—C8—C9	111.5 (2)	C3'—C2'—C1'	109.9 (2)
C7—C8—C26	106.91 (19)	C3'—C2'—H2'1	109.7
C9—C8—C26	110.50 (18)	C1'—C2'—H2'1	109.7
C7—C8—C14	109.20 (18)	C3'—C2'—H2'2	109.7
C9—C8—C14	108.69 (18)	C1'—C2'—H2'2	109.7
C26—C8—C14	110.02 (19)	H2'1—C2'—H2'2	108.2
C11—C9—C8	108.81 (19)	C2'—C3'—C4'	113.7 (3)
C11—C9—C10	114.66 (18)	C2'—C3'—H3'1	108.8
C8—C9—C10	116.76 (18)	C4'—C3'—H3'1	108.8
C11—C9—H9	105.1	C2'—C3'—H3'2	108.8
C8—C9—H9	105.1	C4'—C3'—H3'2	108.8
C10—C9—H9	105.1	H3'1—C3'—H3'2	107.7
C25—C10—C1	106.87 (19)	C5'—C4'—C3'	115.4 (3)
C25—C10—C5	113.51 (19)	C5'—C4'—H4'1	108.4
C1—C10—C5	107.37 (19)	C3'—C4'—H4'1	108.4
C25—C10—C9	113.08 (19)	C5'—C4'—H4'2	108.4
C1—C10—C9	109.20 (18)	C3'—C4'—H4'2	108.4
C5—C10—C9	106.63 (18)	H4'1—C4'—H4'2	107.5
O1—C11—C12	109.2 (2)	C4'—C5'—C6'	112.2 (2)
O1—C11—C9	113.1 (2)	C4'—C5'—H5'1	109.2
C12—C11—C9	113.08 (19)	C6'—C5'—H5'1	109.2
O1—C11—H11	107.0	C4'—C5'—H5'2	109.2
C12—C11—H11	107.0	C6'—C5'—H5'2	109.2
C9—C11—H11	107.0	H5'1—C5'—H5'2	107.9
C13—C12—C11	127.4 (2)	C7'—C6'—C5'	116.1 (2)
C13—C12—H12	116.3	C7'—C6'—H6'1	108.3
C11—C12—H12	116.3	C5'—C6'—H6'1	108.3
C12—C13—C18	119.4 (2)	C7'—C6'—H6'2	108.3
C12—C13—C14	119.8 (2)	C5'—C6'—H6'2	108.3
C18—C13—C14	120.69 (19)	H6'1—C6'—H6'2	107.4
C13—C14—C15	112.2 (2)	C6'—C7'—C8'	112.2 (2)
C13—C14—C27	106.24 (19)	C6'—C7'—H7'1	109.2
C15—C14—C27	106.42 (19)	C8'—C7'—H7'1	109.2
C13—C14—C8	109.24 (18)	C6'—C7'—H7'2	109.2
C15—C14—C8	110.61 (19)	C8'—C7'—H7'2	109.2
C27—C14—C8	112.09 (19)	H7'1—C7'—H7'2	107.9
C16—C15—C14	114.1 (2)	C9'—C8'—C7'	114.6 (2)
C16—C15—H15A	108.7	C9'—C8'—H8'1	108.6
C14—C15—H15A	108.7	C7'—C8'—H8'1	108.6
C16—C15—H15B	108.7	C9'—C8'—H8'2	108.6
C14—C15—H15B	108.7	C7'—C8'—H8'2	108.6
H15A—C15—H15B	107.6	H8'1—C8'—H8'2	107.6
C15—C16—C17	112.8 (2)	C8'—C9'—C10'	113.4 (2)
C15—C16—H16A	109.0	C8'—C9'—H9'1	108.9
C17—C16—H16A	109.0	C10'—C9'—H9'1	108.9
C15—C16—H16B	109.0	C8'—C9'—H9'2	108.9
C17—C16—H16B	109.0	C10'—C9'—H9'2	108.9
H16A—C16—H16B	107.8	H9'1—C9'—H9'2	107.7
C22—C17—C16	111.3 (2)	C9'—C10'—C11'	113.8 (2)
C22—C17—C28	107.1 (2)	C9'—C10'—H10A	108.8
C16—C17—C28	109.9 (2)	C11'—C10'—H10A	108.8
C22—C17—C18	111.0 (2)	C9'—C10'—H10B	108.8
C16—C17—C18	107.82 (19)	C11'—C10'—H10B	108.8
C28—C17—C18	109.7 (2)	H10A—C10'—H10B	107.7
C13—C18—C17	110.3 (2)	C10'—C11'—C12'	113.7 (2)
C13—C18—C19	114.29 (19)	C10'—C11'—H11A	108.8
C17—C18—C19	112.55 (19)	C12'—C11'—H11A	108.8
C13—C18—H18	106.4	C10'—C11'—H11B	108.8
C17—C18—H18	106.4	C12'—C11'—H11B	108.8
C19—C18—H18	106.4	H11A—C11'—H11B	107.7
C29—C19—C20	111.1 (2)	C13'—C12'—C11'	112.2 (2)
C29—C19—C18	111.5 (2)	C13'—C12'—H12A	109.2
C20—C19—C18	110.7 (2)	C11'—C12'—H12A	109.2
C29—C19—H19	107.8	C13'—C12'—H12B	109.2
C20—C19—H19	107.8	C11'—C12'—H12B	109.2
C18—C19—H19	107.8	H12A—C12'—H12B	107.9
C21—C20—C30	109.6 (2)	C12'—C13'—C14'	114.9 (2)
C21—C20—C19	110.1 (2)	C12'—C13'—H13A	108.5
C30—C20—C19	113.1 (2)	C14'—C13'—H13A	108.5
C21—C20—H20	108.0	C12'—C13'—H13B	108.5
C30—C20—H20	108.0	C14'—C13'—H13B	108.5
C19—C20—H20	108.0	H13A—C13'—H13B	107.5
C22—C21—C20	111.0 (2)	C15'—C14'—C13'	112.8 (2)
C22—C21—H21A	109.4	C15'—C14'—H14A	109.0
C20—C21—H21A	109.4	C13'—C14'—H14A	109.0
C22—C21—H21B	109.4	C15'—C14'—H14B	109.0
C20—C21—H21B	109.4	C13'—C14'—H14B	109.0
H21A—C21—H21B	108.0	H14A—C14'—H14B	107.8
C21—C22—C17	114.3 (2)	C14'—C15'—C16'	114.3 (2)
C21—C22—H22A	108.7	C14'—C15'—H15C	108.7
C17—C22—H22A	108.7	C16'—C15'—H15C	108.7
C21—C22—H22B	108.7	C14'—C15'—H15D	108.7
C17—C22—H22B	108.7	C16'—C15'—H15D	108.7
H22A—C22—H22B	107.6	H15C—C15'—H15D	107.6
C4—C23—H23A	109.5	C15'—C16'—H16'A	109.5
C4—C23—H23B	109.5	C15'—C16'—H16'B	109.5
H23A—C23—H23B	109.5	H16'A—C16'—H16'B	109.5
C4—C23—H23C	109.5	C15'—C16'—H16'C	109.5
H23A—C23—H23C	109.5	H16'A—C16'—H16'C	109.5
H23B—C23—H23C	109.5	H16'B—C16'—H16'C	109.5
C4—C24—H24A	109.5		
			
C10—C1—C2—C3	−58.1 (3)	C7—C8—C14—C13	178.74 (18)
C1'—O2—C3—C2	91.8 (3)	C9—C8—C14—C13	56.9 (2)
C1'—O2—C3—C4	−143.6 (2)	C26—C8—C14—C13	−64.2 (2)
C1—C2—C3—O2	179.05 (19)	C7—C8—C14—C15	−57.3 (2)
C1—C2—C3—C4	58.7 (3)	C9—C8—C14—C15	−179.17 (19)
O2—C3—C4—C23	−47.3 (3)	C26—C8—C14—C15	59.7 (2)
C2—C3—C4—C23	73.5 (3)	C7—C8—C14—C27	61.3 (2)
O2—C3—C4—C24	70.0 (2)	C9—C8—C14—C27	−60.6 (2)
C2—C3—C4—C24	−169.2 (2)	C26—C8—C14—C27	178.31 (19)
O2—C3—C4—C5	−173.05 (19)	C13—C14—C15—C16	−36.2 (3)
C2—C3—C4—C5	−52.2 (3)	C27—C14—C15—C16	79.6 (2)
C3—C4—C5—C6	−178.62 (19)	C8—C14—C15—C16	−158.42 (19)
C23—C4—C5—C6	57.2 (3)	C14—C15—C16—C17	53.3 (3)
C24—C4—C5—C6	−63.3 (3)	C15—C16—C17—C22	175.0 (2)
C3—C4—C5—C10	50.0 (3)	C15—C16—C17—C28	56.6 (3)
C23—C4—C5—C10	−74.2 (3)	C15—C16—C17—C18	−63.0 (3)
C24—C4—C5—C10	165.3 (2)	C12—C13—C18—C17	136.7 (2)
C4—C5—C6—C7	160.3 (2)	C14—C13—C18—C17	−45.3 (3)
C10—C5—C6—C7	−64.5 (2)	C12—C13—C18—C19	−95.3 (3)
C5—C6—C7—C8	56.4 (3)	C14—C13—C18—C19	82.7 (3)
C6—C7—C8—C9	−45.0 (3)	C22—C17—C18—C13	178.59 (19)
C6—C7—C8—C26	75.8 (2)	C16—C17—C18—C13	56.5 (2)
C6—C7—C8—C14	−165.16 (19)	C28—C17—C18—C13	−63.2 (3)
C7—C8—C9—C11	175.71 (19)	C22—C17—C18—C19	49.6 (3)
C26—C8—C9—C11	57.0 (2)	C16—C17—C18—C19	−72.5 (3)
C14—C8—C9—C11	−63.9 (2)	C28—C17—C18—C19	167.8 (2)
C7—C8—C9—C10	44.0 (2)	C13—C18—C19—C29	54.9 (3)
C26—C8—C9—C10	−74.8 (2)	C17—C18—C19—C29	−178.3 (2)
C14—C8—C9—C10	164.42 (17)	C13—C18—C19—C20	179.0 (2)
C2—C1—C10—C25	−70.0 (3)	C17—C18—C19—C20	−54.1 (3)
C2—C1—C10—C5	52.1 (3)	C29—C19—C20—C21	−178.1 (2)
C2—C1—C10—C9	167.35 (19)	C18—C19—C20—C21	57.5 (3)
C6—C5—C10—C25	−65.6 (3)	C29—C19—C20—C30	−55.2 (3)
C4—C5—C10—C25	67.2 (3)	C18—C19—C20—C30	−179.5 (2)
C6—C5—C10—C1	176.55 (19)	C30—C20—C21—C22	177.1 (2)
C4—C5—C10—C1	−50.7 (3)	C19—C20—C21—C22	−57.9 (3)
C6—C5—C10—C9	59.6 (2)	C20—C21—C22—C17	55.4 (3)
C4—C5—C10—C9	−167.66 (19)	C16—C17—C22—C21	69.4 (3)
C11—C9—C10—C25	−54.3 (3)	C28—C17—C22—C21	−170.5 (2)
C8—C9—C10—C25	74.7 (2)	C18—C17—C22—C21	−50.7 (3)
C11—C9—C10—C1	64.6 (2)	C3—O2—C1'—O3	5.6 (4)
C8—C9—C10—C1	−166.46 (19)	C3—O2—C1'—C2'	−172.8 (2)
C11—C9—C10—C5	−179.70 (19)	O3—C1'—C2'—C3'	−79.7 (3)
C8—C9—C10—C5	−50.7 (2)	O2—C1'—C2'—C3'	98.7 (3)
C8—C9—C11—O1	162.55 (19)	C1'—C2'—C3'—C4'	173.8 (3)
C10—C9—C11—O1	−64.6 (2)	C2'—C3'—C4'—C5'	73.9 (4)
C8—C9—C11—C12	37.8 (3)	C3'—C4'—C5'—C6'	176.2 (3)
C10—C9—C11—C12	170.67 (18)	C4'—C5'—C6'—C7'	−177.6 (3)
O1—C11—C12—C13	−132.5 (3)	C5'—C6'—C7'—C8'	176.1 (3)
C9—C11—C12—C13	−5.7 (3)	C6'—C7'—C8'—C9'	−179.5 (3)
C11—C12—C13—C18	177.1 (2)	C7'—C8'—C9'—C10'	175.0 (2)
C11—C12—C13—C14	−0.9 (4)	C8'—C9'—C10'—C11'	69.4 (3)
C12—C13—C14—C15	−147.9 (2)	C9'—C10'—C11'—C12'	177.2 (2)
C18—C13—C14—C15	34.1 (3)	C10'—C11'—C12'—C13'	179.0 (3)
C12—C13—C14—C27	96.2 (2)	C11'—C12'—C13'—C14'	176.9 (2)
C18—C13—C14—C27	−81.7 (2)	C12'—C13'—C14'—C15'	174.7 (3)
C12—C13—C14—C8	−24.9 (3)	C13'—C14'—C15'—C16'	177.1 (3)
C18—C13—C14—C8	157.2 (2)		

### Hydrogen-bond geometry (Å, °)

**Table:**

*D*—H···*A*	*D*—H	H···*A*	*D*···*A*	*D*—H···*A*
O1—H1O···O3^i^	0.79 (4)	2.13 (4)	2.886 (3)	161 (3)

Symmetry codes: (i) −*x*+1, *y*−1/2, −*z*+1.
